# Pulmonary cryptococcosis in the setting of immunosuppression by methylprednisolone monotherapy for oral pemphigus: a case report and literature review

**DOI:** 10.3389/fmed.2025.1569949

**Published:** 2025-07-02

**Authors:** Hongxia Mei, Pengchen Bao, Yongxu Wang, Zhijing Wei, Qin Yang, Chen Chen, Yakai Sun, Xinming Su, Jian Kang, WenYang Li

**Affiliations:** ^1^Respiratory and Critical Care Department, The First Hospital of China Medical University, Shenyang, China; ^2^China Medical University, Shenyang, China

**Keywords:** pulmonary cryptococcosis, pemphigus vulgaris, corticosteroids, antifungal therapy, immunity

## Abstract

*Cryptococcosis* is an opportunistic and potentially fatal fungal infectious disease. Pemphigus diseases are characterized by blistering of the cutaneous and mucous membranes. We report a case of pulmonary *cryptococcosis* (PC) following methylprednisolone treatment for pemphigus vulgaris. Additionally, we analyzed a case series of PC infections recorded in PUBMED from 2013 to 2023. A total of 229 cases of PC were included. The median age was 54 years, with 66.4% of patients being male. Those with previous use of corticosteroids or immunosuppressives accounted for 38.4% of cases. Underlying conditions included solid organ transplantations (25.7%), respiratory diseases (6.6%), malignant tumors (6.1%), rheumatoid arthritis (5.7%), hematological malignancies (4.4%), among others. The main source of infection was exposure to birds, poultry, and their feces (12.7%). *Cryptococcus neoformans* was most frequently isolated (76.4%). Overall mortality was 14.8%. Previous use of corticosteroids or immunosuppressants was a risk factor for disseminated *cryptococcus* (*p* < 0.05). Age, underlying disease, dissemination, and no antifungal therapy were independently associated with increased mortality (*p* < 0.05). Co-occurrence of pemphigus and PC is rare. Prompt diagnosis and appropriate treatment of PC are essential to prevent fatal consequences. Corticosteroids or immunosuppressive therapy are associated with the development of disseminated cryptococcal infection. Age, underlying disease, and dissemination are related to increased mortality. Timely antifungal therapy can improve prognosis.

## 1 Introduction

Pulmonary cryptococcosis (PC) is a lethal but underdiagnosed opportunistic invasive mycosis in both immunocompromised and immunocompetent patients (1). The main human pathogens are *Cryptococcus neoformans* (*C. neoformans*) and *Cryptococcus gattii* (*C. gattii*), which have a worldwide distribution and are usually found in soil and bird excreta (2, 3). The lung and central nervous system are the main sites of Cryptococcus infection, followed by the skin, muscles, soft tissue, joints, bones, liver, kidneys, and other organs (4, 5). *Cryptococcosis* is more commonly seen in immunocompromised (IC) hosts; for instance, HIV/AIDS accounted for more than 80% of *Cryptococcosis* cases worldwide in the mid-1980 s (6). Recently, the incidence of HIV-associated *Cryptococcosis* has decreased significantly in most developed nations, with the widespread implementation of successful antiretroviral therapy (7). HIV-negative individuals have become the main infected population of *Cryptococcosis* (8). Among non-HIV patients, PC is the most common non-CNS location (9). Recently, pulmonary fungal infections have been reported to be accompanied by coronavirus disease 2019 (COVID-19) infections, suggesting that fungi may be the next cause of medical burden following COVID-19 (10).

Pemphigus vulgaris (PV), the most common and representative subtype of pemphigus, is a classic organ-specific human autoimmune disease with a poor prognosis in the absence of drug therapy. Its pathogenesis is due to pathogenic autoantibody reactions to the two desmogleins [desmoglein 1 (Dsg1) and desmoglein 3 (Dsg3)], which are characterized by blisters and erosions of the oral mucosa, with or without skin involvement (11). Corticosteroids are the first choice of treatment for pemphigus. However, long-term use of Corticosteroids can lead to compromised immunity (12, 13).

In this study, we reported a rare case of PC in an HIV-negative patient with pemphigus vulgaris, which had been treated with long-term methylprednisolone. This case deserves further discussion on the difficulty of managing two potentially mortal diseases presented simultaneously and highlights the importance of determining the priority of disease treatment. In addition, we reviewed studies from the past 10 years regarding PC in HIV-negative cases and discussed its pathogenesis, risk factors, and prognosis, so as to promote the standardized diagnosis and treatment of PC in HIV-negative patients.

## 2 Case report

**Figure 1:**
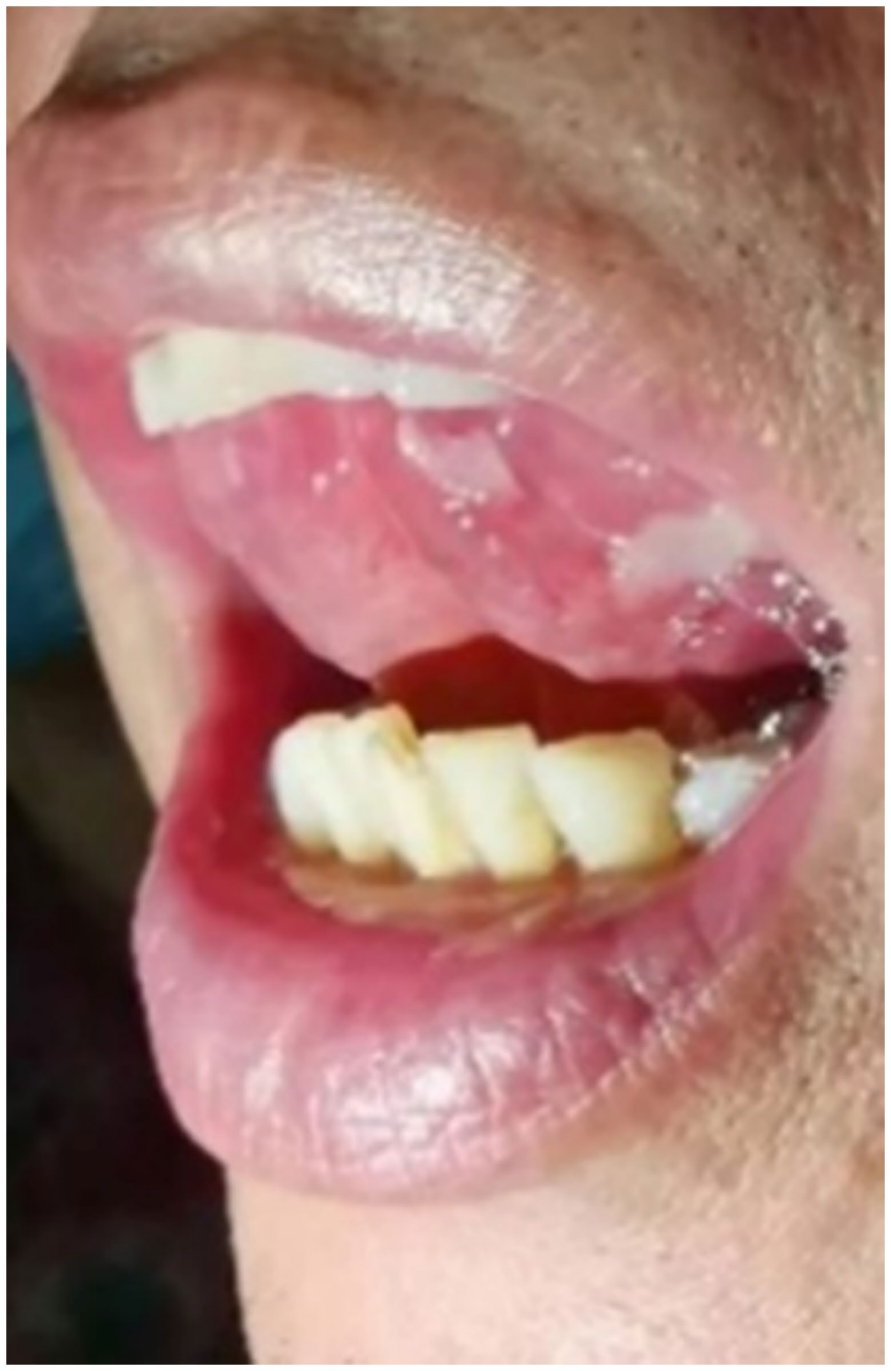
The patient has recurrent ulcers on the ventral side of the tongue.

**Figure 2:**
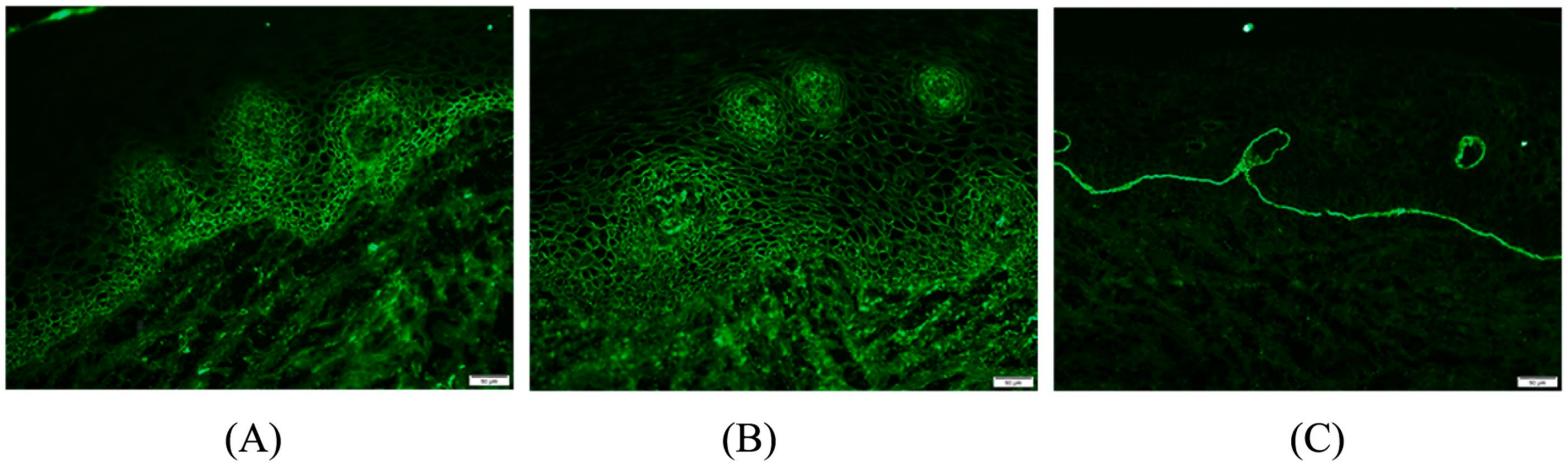
Oral buccal mucosal histopathological biopsy and immunofluorescence: **(A)** Direct immunofluorescence staining of pemphigus vulgaris; **(B,C)** IgG, C3 band deposition at the junction of the epidermis, negative for IgA and IgM.

A 65-year-old man suffered from repeated mouth ulcers for 2 years since July 2018 (Figure 1). His main symptoms involved the oral mucosa with loose blisters that rupture easily, accompanied by intense pain, especially inside the cheeks. The patient had a history of hypertension for 34 years, the highest pressure was 170/100 mmHg, and he orally took a nifedipine sustained-release tablet 10 mg/day. Buccal mucosal pathological biopsy revealed that IgG and C3 banded deposits were at the dermo-epidermal junction, with IgA and IgM negative (Figures 2A–C). Rapid plasma reagin (RPR) was negative, and pemphigus-related antibody test results were as follows: Dsg1*28.6 U/mL, Dsg3*126.9 U/mL, anti-BP 180 antibody*13.7 U U/ml, bullous pemphigoid specific antibody BPAG (BP 230)*4.0RU/ml. These were consistent with the diagnosis of oral pemphigoid. Thereafter, he was treated with oral methylprednisolone, with an initial dose of 32 mg/day. Then it had been tapered over the preceding 8 months to 8 mg/day as his oral symptoms were relieved. For the following 3 months, the patient took methylprednisolone (8 mg, every other day) as maintenance treatment. Triamcinolone acetonide gargle was also given three times a day.

**Figure 3:**
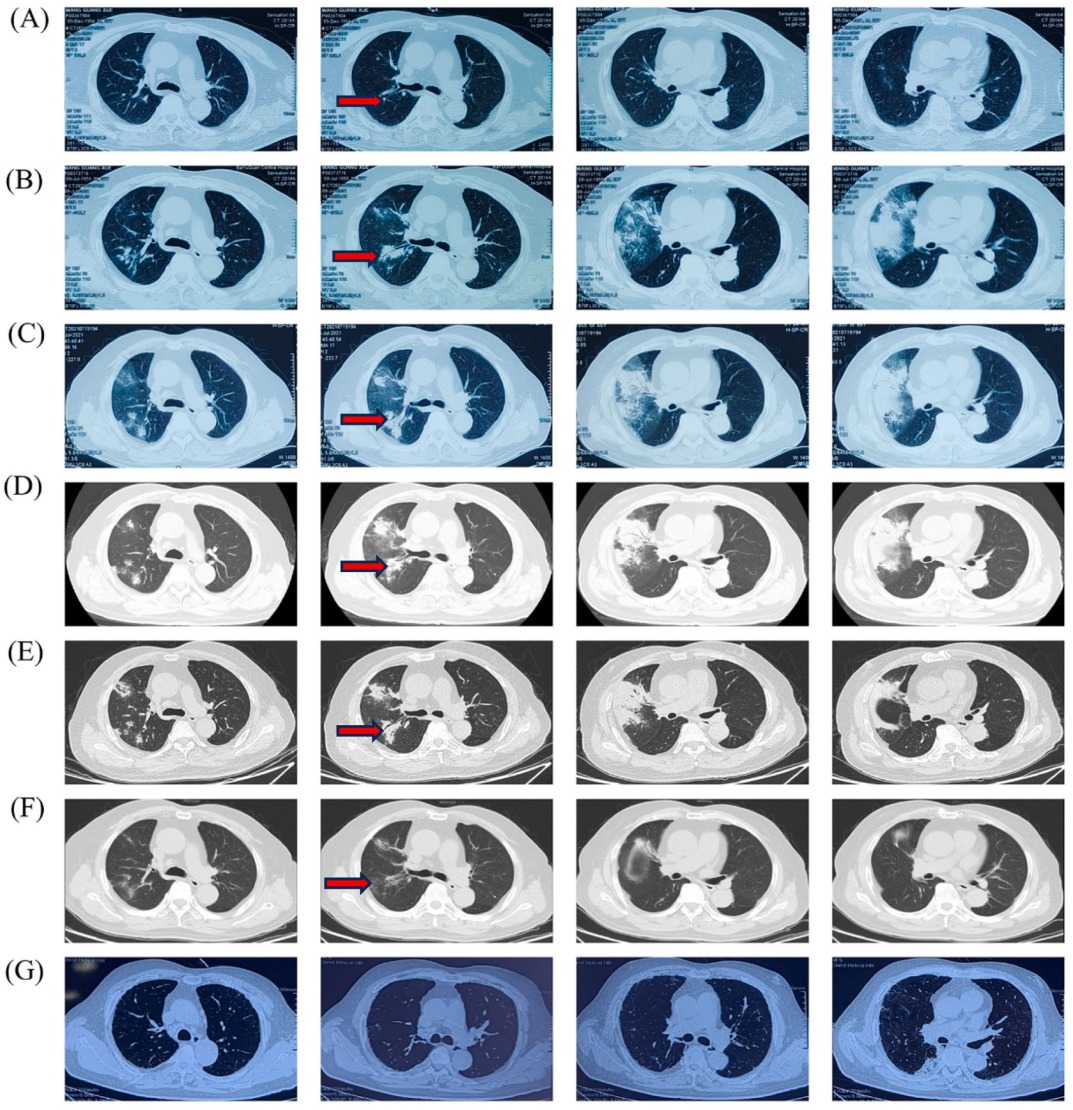
Timeline of the patient’s Chest HRCT imaging scan: **(A)** 15 June 2021, **(B)** 8 July 2021, **(C)** 19 July 2021, **(D)** 26 July 2021, **(E)** 2 August 2021, **(F)** 11 September 2021, **(G)** 4 July 2022.

**Figure 4:**
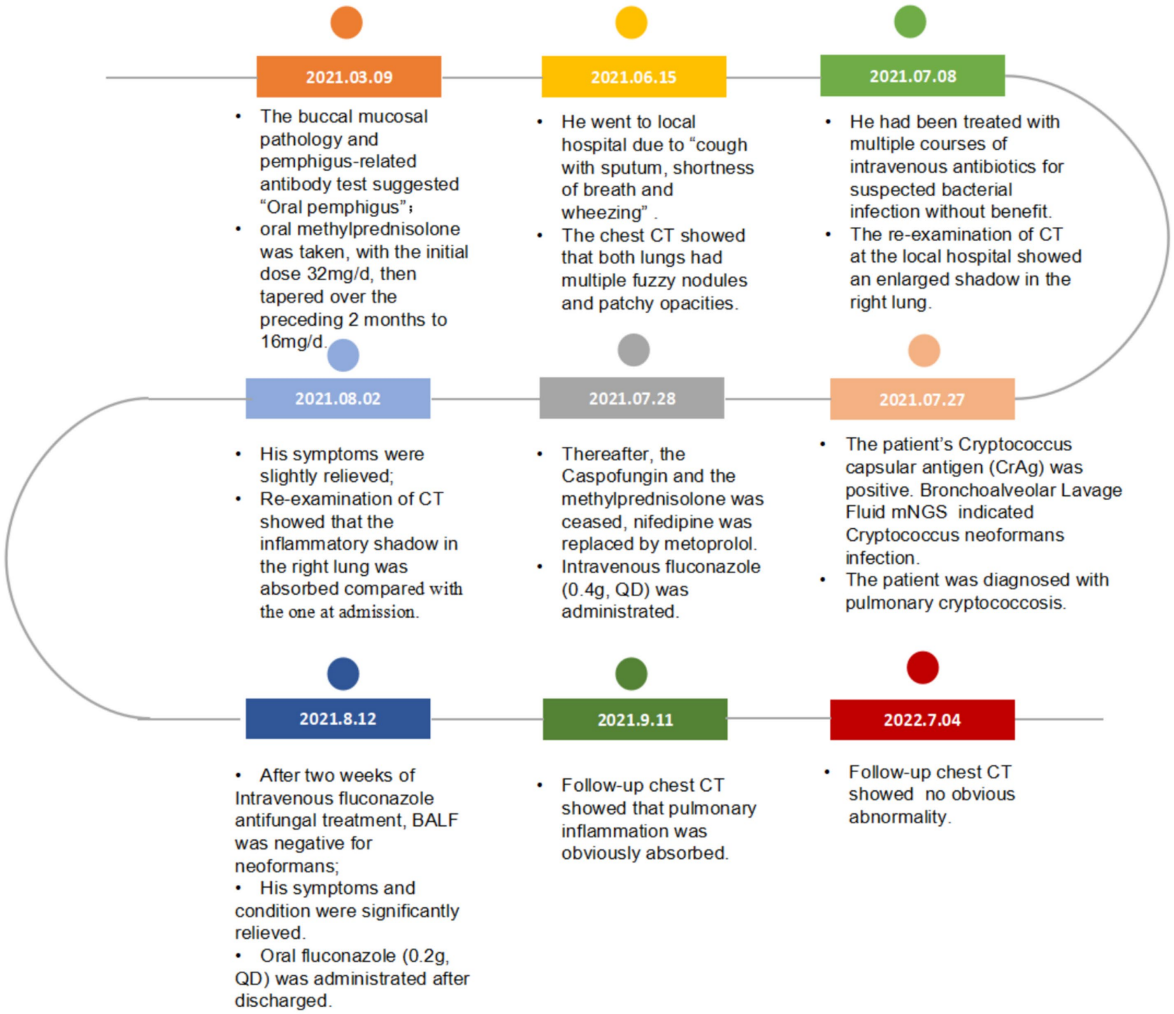
Timeline of the reported case.

**Figure 5:**
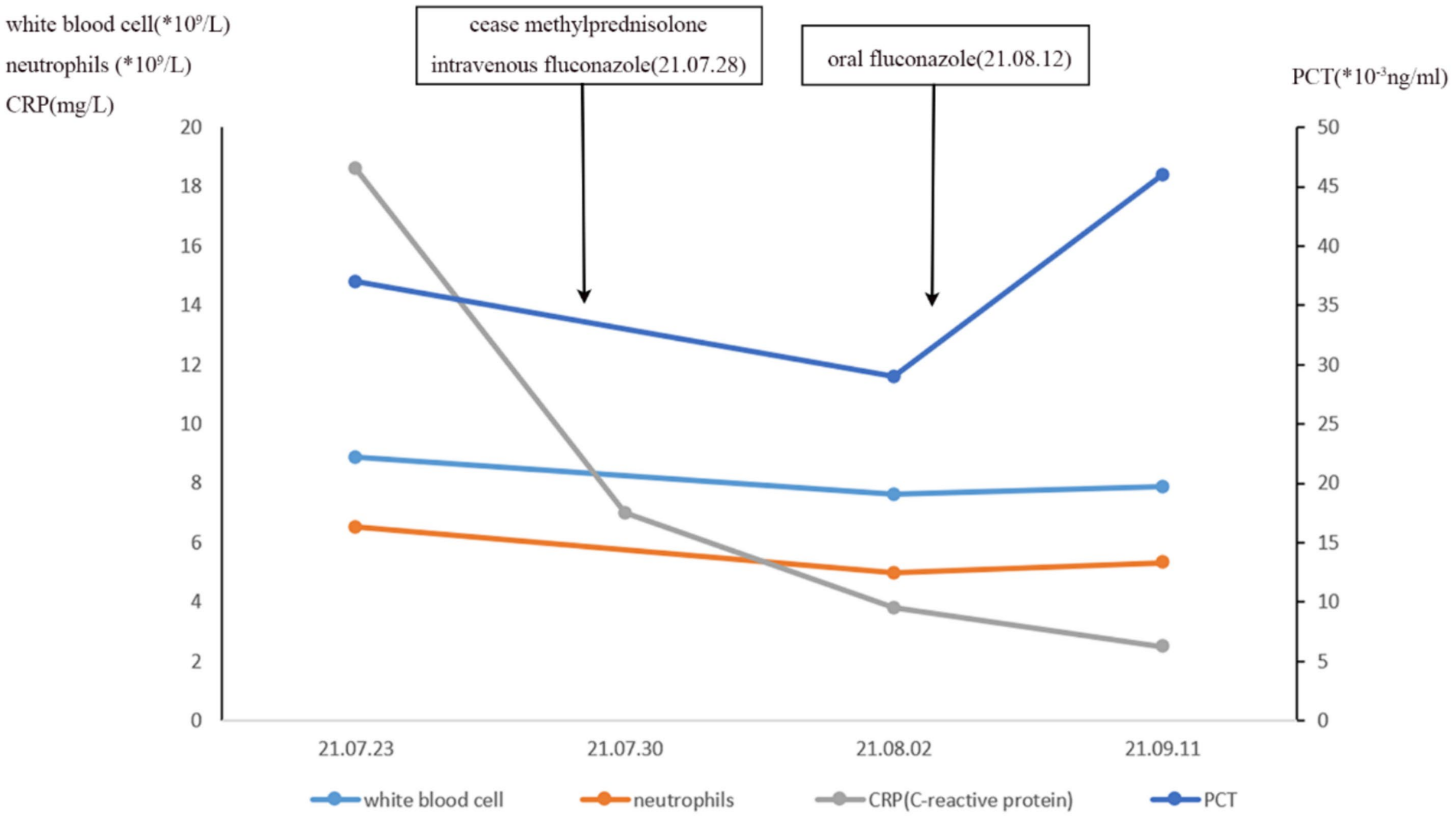
Effect of fluconazole on hematological parameter.

After 5 months of methylprednisolone treatment, the patient developed fever and asthma-like symptoms after exposure to the aerosolized excreta while his neighbor downstairs was cleaning his cockatiel’s cage. Physical examination revealed wheezing sounds from both lungs, more prominent in the right lung. Pulmonary CT showed that both lungs had multiple fuzzy nodules and patchy opacities, with the right lung more prominent (Figure 3A). The pulmonary function test results were as follows: FVC 56.9 L, FEV1/FVC 85.6%, FEV_1pre_ % 62.2, FEV_1after_ % 50.2, bronchodilation test positive. DLCO 88.6%. The laboratory findings were as follows: blood routine: white blood cell count 8.88 × 10^9^/L, neutrophils 6.52 × 10^9^/L, neutrophil ratio 73.4%, hemoglobin 115 g/L; C-reactive protein (CRP) 18.60 mg/L; procalcitonin 0.037 ng/mL; Fungal culture: negative. T cell subtype: CD4(+) T cell count: 261cells/ mm^3^, CD8(+) T cell count 378 cells/ mm^3^. The other tests (liver and renal function) were normal. There was no improvement of the above symptoms following 3 weeks of anti-infective treatment, but a progression of right lung lesions (Figure 3B). After that, the sputum culture results returned “*Candida albicans* ++” infection, and caspofungin antifungal treatment was given. Two weeks later, lung CT was reexamined every 1–2 weeks (Figures 3C–E). Bronchoscopy and alveolar lavage were performed and showed that the bronchoscope and each bronchial cavity were unobstructed, the mucous membrane was smooth, and no obstruction or stenosis was seen. Alveolar lavage fluid (BALF) was tested for general bacterial culture, fungal culture, acid-fast staining, periodic acid-Schiff (PAS) staining, metagenomic next-generation sequencing (mNGS), and other pathogenic examinations. BALF mNGS test (Table 1) reported *Cryptococcus neoformans* (Array Number 19), *Aspergillus flavus* (Array Number 1). Cryptococcus capsular antigen (CrAg) with the colloidal gold method tested with serum was positive (the titer is 35.04). Both the 1,3-*β*-d-glucan (G) test and the galactomannan (GM) test, as well as the PAS staining and acid-fast staining, were negative. BALF mycobacterial rpoB gene (fluorescent PCR): negative. Then, the patient was diagnosed with proven PC. The patient did not show any neurological symptoms, and cerebrospinal fluid was negative for both cryptococcus India ink staining and mNGS. Thereafter, both caspofungin and methylprednisolone were ceased, and intravenous fluconazole (0.4 g, QD) was started. The whole treatment process is shown in Figure 4. After a 2-week treatment, BALF was negative for *C. neoformans*, and the patient’s symptoms and infiltrates in both lungs (Figures 3E) were all greatly improved. After 1 month, the lung CT (Figures 3F, 5) was re-examined in the outpatient clinic: C-reactive protein (CRP) 2.5 mg/L, procalcitonin 0.029 ng/mL, CD4(+) T cell count 779 cells/mm^3^, and CD8(+) T cell count 750 cells/ mm^3^. The patient received oral fluconazole 400 mg/day for 6 months. At the time this case was reported, the patient was followed up for 1 year, and there was no recurrence of cryptococcal infection (Figure 3G).

**Table 1:** Metagenomics next-generation sequencing (mNGS) data.

Genus		Species			
Name	Array number	Name	Array number	Coverage%	Relative abundance%
Cryptococcus	19	*Cryptococcus neoformans*	19	2,249 bp 0.01%	1.10%
Quality-qualified array number: 15037229 (96.66%)					

## 3 Materials and methods

### 3.1 Literature search

Case reports of PC, published in English, Spanish, and French, from 2013 through 2023, were retrieved using the PubMed bibliographic database (U. S. National Library of Medicine, Bethesda, MD). The words included “pulmonary,” “*Cryptococcosis*,” “cryptococcal,” and “pneumonia.” “Cryptococcal infection” was used as a keyword in various combinations, with or without using the Boolean operators ‘AND’ to combine them. After a thorough manual scanning of the bibliography listed in the selected articles and excluding HIV-infected patients, 204 relevant articles were obtained in this review, including 229 patients with pulmonary cryptococcal infection.

### 3.2 Dataset development

Variables in disease development were collected through a manual literature review: The continuous variable was age, and the categorical variables were sex, infection site, causative pathogens, dissemination, microbiome, underlying disease, diagnosis, treatment, and outcome.

### 3.3 Statistical analysis

SPSS (Statistical Package for Social Sciences for Windows) version 26 was used. A two-sided *p*-value of <0.05 was initially considered significant. Categorical variables were compared using x^2^ analysis or Fisher’s exact test. For the estimation of predictors of outcome, univariate analysis was performed. Variables that were entered into the univariate analysis included age>54 years, gender, underlying disease, dissemination, no antifungal therapy, previous corticosteroid or immunosuppressive therapy, and pathogens. Then, variables with a statistically significant relationship, as well as a few considered important in the previous literature, were used to construct a multivariate analysis using logistic regression analysis.

### 3.4 Ethics

Ethical approval was not required for the literature review. Written informed consent was obtained from the patients for publication of the original cases.

## 4 Results

### 4.1 Demographics

A total of 229 cases of PC were included. The median age was 54 [interquartile range (IQR) 25]. There was a significant male predominance, with 152 men (66.4%), 69 women (30.1%), and 8 patients (3.5%) with unspecified gender.

### 4.2 Source of infection

Among the enrolled 229 patients, the possible or confirmed source of pulmonary cryptococcal infection was counted (Table 2), 169 patients (73.8%) denied contact or did not mention in the case to remove the suspected source of infection, 29 patients (12.7%) had contact with birds and poultry and guano, 5 of the patients had contact with poultry, 3 had casual contact with pigeon feces, and 21 had kept or disengaged pigeons or parrots. In total, 4 patients (1.7%) had contact with trees, one was scratched by an olive tree, and one patient was scalded by a burning tree. A total of 7 patients (3.1%) were involved in farm work (one also reported horse contact). Additionally, occupational or environmental exposures included: 1 forest ranger (0.4%), 2 gardeners (0.9%), 1 construction worker (0.4%), 8 people (3.5%) with tourism history, 4 people (1.7%) residing in rural areas, and 1 patient (0.4%) exposed to soil during home construction. History of overseas residence was also reported by 3 people (1.3%).

**Table 2:** Exposure factors of cryptococcal infection (possible or confirmed).

Exposure factors of cryptococcal infection (possible or confirmed)	Case (%)
History of exposure to birds, poultry, or guano	29 (12.7)
History of tree exposure	4 (1.7)
Work on a farm	7 (3.1)
Ranger of forest	1 (0.4)
The gardener	2 (0.9)
Construction worker	1 (0.4)
History of tourism	8 (3.5)
History of rural residence	4 (1.7)
Construction of houses	1 (0.4)
History of overseas residence	3 (1.3)
Other (nothing special or not mentioned)	169 (73.8)
Total amount	229

### 4.3 Underlying diseases

In total, 77 patients (33.6%) had no previous disease history, 152 patients had underlying diseases (Table 3), 22 patients (9.6%) had organ transplantation, including 2 lung transplant patients, 1 liver transplant patient, and 19 kidney transplant patients; 15 patients (6.6%) had respiratory diseases, 5 had chronic obstructive pulmonary disease (COPD), 3 had asthma, 5 had pneumonia, and 2 had acute respiratory distress syndrome (ARDS) secondary to pneumonia. Of the 14 (6.1%) malignant tumors, 6 were lung cancer, 2 were ectopic adrenocorticotropin-secreting carcinoid, 1 was gastric cancer, 1 was prostate cancer, 1 was oral cancer, 1 was breast cancer, 1 was pancreatic cancer, and 1 was bladder cancer; 13 patients (5.7%) had rheumatoid arthritis. There were 10 cases (4.4%) of hematological malignancies, 1 case of chronic lymphocytic leukemia; 3 cases of acute lymphoblastic leukemia, 1 case of multiple myeloma; 1 case of plasmacytoma, 1 case of diffuse large B-cell lymphoma; 10 cases (4.4%) of hepatitis/liver cirrhosis, 5 patients had hepatitis B, 1 had hepatitis C, and 4 had cirrhosis; 7 cases (3.1%) of tuberculosis; 7 cases (3.1%) of diabetic nephropathy or nephropathy, including 2 cases of diabetic nephropathy; 6 cases (2.6%) of Crohn’s disease; 5 cases (2.2%) of systemic lupus erythematosus; 5 cases (2.2%) of benign tumors included two yolk sac tumors, one pituitary adenoma, one uterine fibroid, and one thymoma; and 5 cases (2.2%) of cardiovascular diseases, including one patient with ischemic cardiomyopathy, two patients with MI, and two patients with atrial fibrillation. Primary myelofibrosis in 3 patients (1.3%), postpartum 3 cases (1.3%), idiopathic Cushing syndrome in 3 patients (1.3%), 2 (0.9%) cases of Castleman disease, acquired alveolar protein deposition disease in 2 cases (0.9%), CD4 lymphocyte reducing disease in 2 cases (0.9%), 2 cases of ulcerative colitis (0.9%), 2 cases of sarcoidosis (0.9%), 2 case of IgG4-RD (0.9%), 1 case of multiple sclerosis (0.4%), 1 case of granulocyte-macrophage colony-stimulating factor (GM-CSF) (0.4%), 1 case of bullous pemphigus (0.4%), 1 case of psoriasis (0.4%), 1 case of gout (0.4%) 1 case of neurosarcoidosis (0.4%), and 1 case of human T-philia (0.4%); 1 case (0.4%) had lymphocytic virus 1 (HTLV-1), 1 case (0.4%) had seronegative symmetrical synovitis with pitting edema syndrome, 1 case (0.4%) had hypercalcemia, 1 case (0.4%) had pancytopenia, 1 case (0.4%) had Sjogren’s syndrome, and 1 case (0.4%) had cerebral infarction.

**Table 3:** Underlying conditions.

Underlying conditions	Case (%)	Mortality (%)
None	77 (33.6)	4/77 (5.2)
Organ transplantation	22 (9.6)	4/22 (9.1)
Respiratory diseases	15 (6.6)	5/15 (33.3)
Malignant tumor	14 (6.1)	4/14 (28.6)
Rheumatoid arthritis	13 (5.7)	4/13 (30.8)
Hematological malignancies	10 (4.4)	–
Hepatitis/Cirrhosis	10 (4.4)	4/10 (40)
Tuberculosis	7 (3.1)	2/7 (28.6)
Diabetic Nephropathy/Nephropathy	7 (3.1)	2/7 (28.6)
Crohn’s Disease	6 (2.6)	–
Systemic lupus erythematosus	5 (2.2)	1/5 (20)
Benign tumors	5 (2.2)	1/5 (20)
Cardiovascular diseases	5 (2.2)	1/5(20)
Primary myelofibrosis	3 (1.3)	1/3 (33.3)
Postpartum	3 (1.3)	–
Cushing’s syndrome	3 (1.3)	–
Acquired pulmonary alveolar proteinosis	2 (0.9)	–
Castleman’s disease	2 (0.9)	–
Idiopathic CD4 lymphopenia	2 (0.9)	–
Ulcerative colitis	2 (0.9)	–
IgG4-RD^a^	2 (0.9)	–
Sarcoidosis	2 (0.9)	–
Bullous pemphigus	1 (0.4)	-
Psoriasis	1 (0.4)	–
Gout	1 (0.4)	–
Neurosarcoidosis	1 (0.4)	–
Multiple sclerosis	1 (0.4)	–
Human T-lymphotropic virus 1 (HTLV-1)	1 (0.4)	1/1 (100)
Seronegative symmetrical synovitis with pitting edema syndrome	1 (0.4)	–
Hypercalcemia	1 (0.4)	–
Pancytopenia	1 (0.4)	–
Sjogren’s syndrome	1 (0.4)	–
Granulocyte-macrophage colony-stimulating factor (GM-CSF)	1 (0.4)	–
Cerebral infarction	1 (0.4)	–
Total amount	229	34

a IgG4-RD, immunoglobulin G4-related disease.

### 4.4 Radiological appearances and diagnosis

#### 4.4.1 Radiological characteristics

Radiological findings were unreported in 70 cases (30.6%). Findings included: nodular shadows (10 cases, 4.4%), multiple nodular shadows (72 cases, 31.4%), nodules with lymph node enlargement (22 cases, 9.6%), void (9 cases, 3.9%), infiltration (11 cases, 4.8%), ground-glass shadow (20 cases, 8.7%), pleural effusion (6 cases, 2.6%), pericardial effusion (1 case, 0.4%), consolidation (5 cases, 2.2%), and air bronchogram (3 cases, 1.3%) (Tables 4, 5). Eighty-two cases (35.8%) showed bilateral lung involvement, 53 cases (23.1%) involved the right lung, and only 24 cases (10.5%) involved the left lung.

**Table 4:** Radiological characteristics.

Radiological appearances	Case (%)
Unknown	70 (30.6)
Solitary nodule	10 (4.4)
Multiple nodules	69 (30.1)
Nodules accompanied by lymphadenopathy	22 (9.6)
void	9 (3.9)
Shadow of infiltration	11 (4.8)
Ground glass shadow	20 (8.7)
Pleural effusion	6 (2.6)
Pericardial effusion	1 (0.4)
Consolidation change	5 (2.2)
Air bronchogram sign	3 (1.3)
Total	229
Pulmonary involvement position	
Unknown	70 (30.6)
Bilateral lungs	82 (35.8)
Right lung	53 (23.1)
Left lung	24 (10.5)
Total	229

**Table 5:** Diagnostic methods.

Diagnostic method(s) used	Case (%)
Unspeciated	2 (0.9)
CT + Histology	63 (27.5)
CT + culture	9 (3.9)
CT + CrAg	9 (3.9)
CT+mNGS^a^	2 (0.9)
FDG-PET+Histology	3 (1.3)
CT + Histology+CrAg	39 (17)
CT + Histology+direct microscopy	1 (0.4)
CT + Histology+direct microscopy+culture	2 (0.9)
CT + Histology+direct microscopy+CrAg	4 (1.7)
CT + Histology+PCR^b^	4 (1.7)
CT + Histology+culture	18 (7.9)
CT + Histology+culture+CrAg	10 (4.4)
CT + Histology+culture+direct microscopy+CrAg	2 (0.9)
CT + Histology+MALDI-TOF MS^c^	3 (1.3)
CT + Histology+culture+PCR	1 (0.4)
CT + Histology+CrAg+PCR	1 (0.4)
CT + culture+CrAg	19 (8.3)
CT + culture+mNGS	2 (0.9)
CT + culture+MALDI-TOF MS	2 (0.9)
CT + culture+PCR	1 (0.4)
CT + CrAg+mNGS	1 (0.4)
CT + CrAg+PCR	2 (0.9)
CT + direct microscopy	2 (0.9)
CT + direct microscopy+CrAg	7 (3.1)
CT + direct microscopy+culture	16 (7)
CT + direct microscopy+culture+CrAg	2 (0.9)
CT + direct microscopy+culture+CrAg +MALDI-TOF MS	1 (0.4)
CT + direct microscopy+CrAg +MALDI-TOF MS + PCR	1 (0.4)
Total	229

a mNGS, metagenomic next-generation sequencing.

b PCR, polymerase chain reaction.

c MALDI- TOF, matrix-assisted laser desorption/ionisation – time of flight (mass spectrometry).

#### 4.4.2 Diagnosis of cryptococcus

The diagnosis of pulmonary cryptococcal infection was complicated; 28 diagnostic procedures were used to diagnose cryptococcal infection. In total, 63 cases (27.5%) were histopathological biopsy after radiological examination, 9 cases (3.9%) were radiological examination only combined with culture, and 9 cases (3.9%) were CT only combined with cryptococcal antigen (CrAg) test. CT combined with fluorodeoxyglucose positron emission tomography (FDG-PET) tuberculosis histopathology was performed in 3 cases (1.3%); CT, histopathology, and cryptococcal antigen examination in 39 cases (17%); and radiology, histopathology, and direct microscopy in 1 case (0.4%). Histopathology, direct microscopy and etiology in 2 cases (0.9%); histopathology, direct microscopy and cryptococcal antigen 4 cases (1.7%); imaging and pathological histology PCR 4 cases (1.7%) combined with molecular detection, imaging and pathological organization combined with the cultivation of 18 cases (7.9%); CT, pathology, culture and cryptococcal antigen were used in 10 cases (4.4%). These four methods, combined with direct microscopic examination in 2 cases (0.9%), PCR was only used in 9 cases (3.5%), and matrix-assisted laser desorption ionization-time of flight mass spectrometry (MALDI-TOF MS) was only used in 7 cases (3.1%). Metagenomic next-generation sequencing (mNGS) was found in only 5 cases (0.9%) (Table 6).

**Table 6:** Classification of pathogens.

Classification of pathogens	Case (%)	Mortality (%)
*C. neoformans*	175 (76.4)	28/175 (16)
*C. gatti*	38 (16.6)	6/38 (15.7)
Unidentified pathogen	16 (7)	0
Total	229	34/229 (14.8)

#### 4.4.3 Classification of pathogens

*Cryptococcus neoformans* (76.4%) was most frequently isolated species, followed by *Cryptococcus gatti* (16.6%) (Table 7). The mortality rate of *C. neoformans* infection was 16%, while that for *C. gattii* infection was 15.7%.

**Table 7:** Previous medication history.

Previous medication history	Case (%)	Disseminated case (%)	Mortality (%)
Unknown	61 (26.6)	12 (12.9)	12/34 (35.3)
Previous corticosteroid or immunosuppressive therapy	88 (38.4)	51 (54.8)	18/34 (52.9)
No medication	80 (34.9)	30 (32.3)	4/34 (11.8)
Total	229	93	34

### 4.5 Disseminated cryptococcus

Pulmonary cryptococcus infection often disseminates to the brain, blood, skin, bone, and other parts of the body. Among 61 cases (26.6%) with unknown medication history, 12 cases (26.6%) disseminated to other tissues (26.6%) and 12 cases (35.3%) died. 88 cases were previously treated with corticosteroids or immunosuppressive agents, 51 cases (54.8%) disseminated, and 18 cases (52.9%) died. Among the 80 cases who did not take the drug, 30 cases (32.3%) disseminated and 4 cases (11.8 %) died (Table 7). Age, gender, underlying diseases, and other variables were included to construct a multivariate logistic regression equation (Table 8). Only pathogens were the independent factor associated with the dissemination of cryptococcus (OR = 0.30, 95%CI = 0.132–0.669, *p* < 0.01) (Table 8). In order to further analyze the occurrence of dissemination in *C. neoformans* infection, we constructed a multivariate logistic regression equation by including variables such as age, sex, underlying diseases, and previous corticosteroid or immunosuppressive therapy (Table 9). Medication history of corticosteroid or immunosuppressive was associated with increased risk of *C. neoformans* dissemination (OR = 2.40, 95%CI = 1.14–5.24, *p* = 0.02) (Table 9).

**Table 8:** Analysis of risk factors for dissemination.

Variables	Dissemination rate%	Univariate analysis		Multivariate analysis	
		*p*-value	OR^a^ (95% CI^b^)	*p*-value	OR (95% CI)
Age>54	43.00%	0.75	0.91(0.53–1.58)	0.46	1.27(0.68–2.35)
Gender	44.80%	0.67	0.89(0.5–1.58)	0.95	1.02(0.55–1.88)
Underlying disease	40.70%	0.17	0.67(0.37–1.18)	0.07	0.50(0.23–1.06)
Previous corticosteroid or immunosuppressive therapy	46.50%	0.53	1.19(0.69–2.07)	0.06	1.95(0.97–3.94)
Classification of pathogens	39.90%	0	0.32(0.15–0.69)	0.00	0.30(0.13–0.67)

a OR, odds ratio.

b CI, confidence intervals.

**Table 9:** Analysis of risk factors for dissemination in *C. neoformans* infection.

Variables	Dissemination rate%	Univariate analysis		Multivariate analysis	
		*p*-value	OR (95% CI)	*p*-value	OR (95% CI)
Age>54	41.10%	0.66	1.15(0.625–2.099)	0.66	1.16(0.60–2.23)
Gender	36.10%	0.80	1.10(0.58–2.1)	0.57	1.21(0.63–2.34)
Underlying disease	30.50%	0.65	0.86(0.44–1.67)	0.86	0.47(0.20–1.12)
Previous corticosteroid or immunosuppressive therapy	44.90%	0.10	1.66(0.91–3.06)	0.02	2.44(1.14–5.24)

### 4.6 Treatments and outcomes

The common antifungal drugs for cryptococcal infection include amphotericin B, flucytosine, voriconazole, fluconazole, and so on (14). The majority of patients survived after treatment, and 8 patients did not receive treatment. Among them, the mortality rate was 12% in cases treated with antifungal therapy and 75% in untreated cases. A total of 6 patients died without treatment because of undetected cryptococcal infection, only 2 patients recovered spontaneously, and 6 patients died due to disease deterioration (Table 10). Age, gender, underlying diseases, and other variables were included to construct a multivariate logistic regression equation (Table 11). As a result, variables including age > 54 years (OR = 4.28, 95% CI = 1.52–12.0, *p* = 0.01), underlying disease (OR = 4.43, 95% CI = 1.14–17.25, *p* = 0.03), dissemination (OR = 11.36, 95% CI = 3.88–33.31, *p* < 0.01) and no antifungal therapy (OR = 28.48, 95% CI = 4.19–193.8, *p* < 0.01) were associated with increased risk of death (Table 11).

**Table 10:** Treatments and outcomes.

Therapy	Case (%)	Mortality (%)
Antifungal therapy	221(96.5)	28/221(12.7)
No antifungal therapy	8(3.5)	6/8(75)
Total	229	34/229(14.8)

**Table 11:** Analysis of risk factors for mortality.

Variables	Death rate (%)	Univariate analysis		Multivariate analysis	
		*p*-value	OR (95% CI)	*p*-value	OR (95% CI)
Age>54	24.0%	0.00	3.25(1.47–7.20)	0.01	4.28(1.52–12.03)
Gender	14.3%	0.89	1.06(0.48–2.32)	0.66	0.81(0.31–2.126)
Underlying disease	20.0%	0.02	3.20(1.18–8.67)	0.03	4.43(1.14–17.25)
Dissemination	29.8%	0.10	1.85(0.89–3.87)	0.00	11.36(3.88–33.31)
No antifungal therapy	75.0%	0.00	8.06(3.17–20.48)	0.00	28.48(4.19–193.87)
Previous corticosteroid or immunosuppressive therapy	20.1%	0.89	0.93(0.36–2.45)	0.52	0.71 (0.25–2.02)
Classification of pathogens	15.7%	0.00	19.07(3.67–99.22)	0.99	0.988(0.31–2.13)

## 5 Discussion

To the best of our knowledge, this is the first analytic attempt at the HIV-negative PC cases reported within the last 10 years. This study helps to further understand the pathogenesis and current situation of *Cryptococcosis* in HIV-negative patients. This case emphasizes the potential complications of immunosuppressive therapy with methylprednisolone for pemphigus vulgaris, and the prompt diagnosis and appropriate treatment are essential to prevent fatal consequences.

Previously, HIV-associated cryptococcal infections have been a major concern, with an estimated global prevalence of cryptococcal antigenemia of 4.4% among HIV patients with CD4^+^ counts <200 cells/μL, corresponding to approximately 179,000 cases in 2020. Of these, 152,000 progressed to cryptococcal meningitis, causing 112,000 deaths, accounting for 19% of AIDS-related mortality globally (15). Recently, increasing attention has also been directed toward cryptococcal infections in HIV-negative patients. This case deserves further discussion on the difficulty of managing two potentially mortal diseases presented simultaneously and highlights the importance of determining the priority of disease treatment. PC is a life-threatening invasive fungal infection caused by the yeast-like capsule of Cryptococcus. It has been documented that some underlying diseases were closely related to cryptococcus infection, such as organ transplantation, malignant tumors, malignant hematological diseases, rheumatoid arthritis, severe diabetes mellitus, and other conditions that impair T-cell-mediated immunity (16, 17). Meanwhile, long-term treatment for some of these diseases includes immunosuppressive agents or corticosteroids are also predisposing factors that could further worsen the prognosis of PC (18). In our case, the patient was HIV-negative and without an underlying condition that renders compromised immunity. However, he was treated with methylprednisone monotherapy for pemphigus vulgaris (19). During treatment for PC, he continued maintenance prednisone therapy for pemphigus vulgaris, as corticosteroids are considered the primary and most accessible treatment for pemphigus (20). However, both pemphigus vulgaris and *Cryptococcosis* are potentially fatal conditions, and treatment of the former may worsen the latter. Complicating the situation further, the patient’s oral ulcers remained persistent and recurrent despite receiving methylprednisolone pulse therapy (initial dose 32 mg/ d) for 5 months. In this case, we noted that the patient was treated with nifedipine extended-release tablets 10 mg/ day orally for antihypertensive treatment. Previous studies have documented that some drugs containing active sulfhydryl groups, such as penicillamine, captopril, clonidine, atenolol, propranolol, Nadolol, etc., can cause decidual or mucosal pemphigus. While some non-sulfhydryl antihypertensive drugs, including nitrendipine and nifedipine, can also cause or aggravate pemphigus vulgaris (21–23). This may explain the recurrence of pemphigus symptoms (oral ulcers, etc.) despite a long-term standardized use of corticosteroids for 5 months. Based on these considerations, we decided to timely replace nifedipine with metoprolol (23.75 mg, QD), then rapidly reduce the dose of methylprednisone until removal. After that, his pemphigus was well controlled, and the prognosis of PC was largely improved following antifungal treatment. It suggests that clinical thinking is critical when deciding whether to reduce or even suspend immunosuppressive regimens when weighing two potentially life-threatening conditions.

In China, immunocompetent patients account for 40% of cases of isolated PC, with a higher proportion of male patients than female patients (24). Globally, there is also a sex-biased distribution, with more men suffering cryptococcal infections than women in both the HIV-positive (4:1 ratio between men and women) and HIV-negative (3:1 ratio between men and women) patients (25). The predominance of male patients in morbidity was also observed in this study. In addition, one has observed that among infected patients, men typically experienced more severe symptoms than women (25), and sex hormones may be implicated as a contributing factor for this sex difference. Some normal immune patients have to be treated with corticosteroids, medications including corticosteroids are a risk factor for pulmonary *Cryptococcosis* (26, 27). Isolated pulmonary disease with rapid progression can occur in the immunocompromised host and is an increasingly important cause of invasive fungal infection (IFI) in immunocompetent patients. One has estimated that there are annually 278,000 invasive cryptococcal infections around the globe (28). However, this is likely a severe underestimate due to under-reporting and coinfections mixed with other more prominent agents, such as tuberculosis.

The consequences of infection with Cryptococcus range from asymptomatic airway colonization to respiratory symptoms of varying severity to disseminated infections that may involve the eye, skin, CNS, and other organs. Progressive pulmonary disease and serious respiratory syndromes are more likely to occur in immunocompromised patients. This study observed that the mortality of PC was 14.8% overall, whereas the death rates increased up to 52.9% among those with previous use of corticosteroid or immunosuppressive therapy. Moreover, among the 34 patients who died, 30 (30 of 34, 88.2%) had underlying diseases and 28 (28 of 34, 82.4%) were confirmed to have cryptococcal dissemination. In addition, the previous use of corticosteroid or immunosuppressive therapy was not an independent factor for death, whereas age > 54 years (OR = 4.28), the existence of underlying disease (OR = 4.43), dissemination (OR = 11.36), and no antifungal treatment (OR = 28.48) were independently associated with increased mortality. Underlying disease is a risk factor for mortality in non-HIV patients, especially those with respiratory disease (33%) and hepatitis/cirrhosis (40%), highlighting the importance of early recognition among these individuals with underlying diseases.

There are over 30 species of Cryptococcus, but only *Cryptococcus neoformans* (*C. neoformans*) and *Cryptococcus gattii* (*C. gattii*) commonly affect animals and humans. In the literature review of this study, *C. neoformans* was found to be most frequently isolated (76.4%). Noteworthy, in immunocompromised patients, those with *C. neoformans* infection are prone to cause central system dissemination and progress into cryptococcal meningitis, both of which are life-threatening and require early detection and timely treatment (29). *C. neoformans* can temporarily colonize the gut of some birds (30, 31). It can also be found in the excreta of asymptomatic birds, contaminated soil, and henhouses, either because it is shed from the bird or because the excreta provides nutrients for organisms to proliferate. Our patient reported a history of exposure to the aerosolized bird excreta. In this study, we observed that 29 patients (12.7%) were exposed to bird feces during the possible exposure history, which is the most common transmission of contamination. After the individual inhales dehydrated yeast cells or spores in the air, these yeast cells or spores pass through the respiratory tract into the deep lung tissue. In immunocompetent hosts, fungal cells are cleared by the immune system, or an asymptomatic latent infection is established. Macrophages are involved in this process (32–34). In immunocompromised patients, Cryptococcosis can cause an intrapulmonary infection with this opportunistic fungus (35). Nevertheless, we also observed that 73.9% of HIV-negative patients with PC do not have a possible exposure history. Previously, cryptococcal dissemination was observed to be associated with underlying diseases such as organ transplantation, malignant tumors, hematological malignancies, and rheumatoid arthritis (36, 37). In this study, there was a statistical difference between *C. neoformans* and *C. gatti* infection for the chance of cryptococcal dissemination (*p* < 0.01, OR = 0.03). Furthermore, we performed an analysis using data with *Cryptococcus neoformans* infection alone, and observed that the previous use of corticosteroids or immunosuppressive therapy is a risk factor for the dissemination of *Cryptococcus* (OR = 2.44).

To date, PC is still underdiagnosed for the limitations in diagnostic tools. Histopathology is the most commonly used diagnostic method for pulmonary cryptococcal infection when sputum and bronchoscopy are fruitless. Clinical manifestations and CT features of PC were diverse and atypical, thus, the diagnosis of PC is challenging (38–40). Pulmonary nodules are the most common radiological feature (69/229, 30.1%), but these are not specific to PC. Serum CrAg tests and mNGS of BALF can facilitate the early detection of *cryptococcosis*. In this case, PC was accurately diagnosed by a combination of CT, serology (CrAg positive), and mNGS of BALF. As reported, mNGS was used for the diagnosis of 5 cases (Table 5), since mNGS has higher sensitivity with a large amount of information, and it can be directly sequenced and identified, which greatly saves detection time and improves diagnostic efficiency. It has unparalleled advantages in the identification of unknown species and pathogens that are difficult to culture (41).

Taking into account all factors, including the host immunosuppression status, antifungal response, and the presence of neurologic complications, physicians may consider induction therapy with amphotericin B and 5-flucytosine, followed by replacement therapy with voriconazole or itraconazole (200 mg) (42). Recent literature recommends the use of fluconazole 400 mg/day orally for 6–12 months in symptomatic or asymptomatic settings (43). In this case, fluconazole (400 mg, QD) was administered intravenously for 14 days during hospitalization, followed by oral fluconazole for 6 months. The patient was followed up for 1 year, and no recurrence of PC was found. Under immunosuppression, the mortality rate of *Cryptococcosis* in HIV-negative patients is high (20%), and the acute mortality after the occurrence of cryptococcal meningoencephalitis can be as high as 35–40%, even without the opportunity for antifungal treatment, so timely diagnosis and medication are particularly important (44–46). In this case, antifungal therapy was timely initiated, so the prognosis is favorable and without cryptococcal meningitis. Timely initiation of antifungal therapy was considered to be critical, especially among patients with advanced age, with underlying disease, and dissemination.

## 6 Conclusion

This case is a unique example of a rare manifestation of PC in the treatment of pemphigus vulgaris with prednisone therapy. Clinical thinking is critical when deciding whether to reduce or even suspend immunosuppressive regimens when weighing two potentially life-threatening conditions. Epidemiological analysis suggests that previous corticosteroid use may promote the development of disseminated cryptococcus. Among HIV-negative patients, age, underlying diseases, and dissemination were associated with a higher risk of mortality, and timely antifungal therapy might improve the prognosis.

## Data Availability

The original contributions presented in the study are included in the article, further inquiries can be directed to the corresponding authors.
